# Identifying the first folded alkylbenzene *via* ultraviolet, infrared, and Raman spectroscopy of pentylbenzene through decylbenzene†
†Electronic supplementary information (ESI) available. See DOI: 10.1039/c7sc02027a


**DOI:** 10.1039/c7sc02027a

**Published:** 2017-05-23

**Authors:** Daniel M. Hewett, Sebastian Bocklitz, Daniel P. Tabor, Edwin L. Sibert III, Martin A. Suhm, Timothy S. Zwier

**Affiliations:** a Department of Chemistry , Purdue University , West Lafayette , IN 47907 , USA . Email: zwier@purdue.edu; b Institut für Physikalische Chemie , Universität Göttingen , Göttingen , Germany; c Department of Chemistry , University of Wisconsin-Madison , Madison , WI 53706 , USA . Email: elsibert@wisc.edu

## Abstract

The conformational preferences of pentyl- through decylbenzene are studied under jet-cooled conditions in the gas phase. Laser-induced fluorescence excitation spectra, fluorescence-dip infrared spectra in the alkyl CH stretch region, and Raman spectra are combined to provide assignments for the observed conformers. Density functional theory calculations at the B3LYP-D3BJ/def2TZVP level of theory provide relative energies and normal mode vibrations that serve as inputs for an anharmonic local mode theory introduced in earlier work on alkylbenzenes with *n* = 2–4. This model explicitly includes anharmonic mixing of the CH stretch modes with the overtones of scissors/bend modes of the CH_2_ and CH_3_ groups in the alkyl chain, and is used to assign and interpret the single-conformation IR spectra. In octylbenzene, a pair of LIF transitions shifted –92 and –78 cm^–1^ from the all-*trans* electronic origin have unique alkyl CH stretch transitions that are fit by the local model to a g1g3g4 conformation in which the alkyl chain folds back over the aromatic ring π cloud. Its calculated energy is only 1.0 kJ mol^–1^ above the all-*trans* global minimum. This fold is at an alkyl chain length less than half that of the pure alkanes (*n* = 18), consistent with a smaller energy cost for the g1 dihedral and the increased dispersive interaction of the chain with the π cloud. Local site frequencies for the entire set of conformers from the local mode model show ‘edge effects’ that raise the site frequencies of CH_2_(1) and CH_2_(2) due to the phenyl ring and CH_2_(*n* – 1) due to the methyl group. The g1g3g4 conformer also shows local sites shifted up in frequency at CH_2_(3) and CH_2_(6) due to interaction with the π cloud.

## Abstract 2

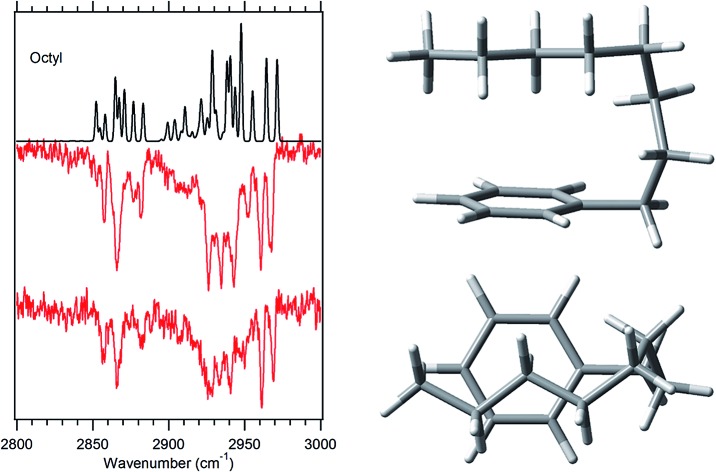
The shortest possible single-chain alkylbenzene to exist in a folded conformation is determined using spectroscopic and theoretical techniques.

## I. Introduction

Alkylbenzenes are principal components of petroleum-based gasoline and diesel fuel, comprising some 20–30% of their chemical composition.^1^ Both single-chain and multiple-chain alkylbenzenes are prevalent, with chains varying in length from methyl groups to ten or more carbon atoms.^2^–^4^ Indeed, decylbenzene has recently been proposed as a surrogate for long-chain alkylbenzenes in diesel fuel.^5^ One of the interesting facets of the presence of these long-chain alkyl groups attached to benzene is that their juxtaposition in a single molecule produces combustion chemistry characteristic of both aromatics and alkanes simultaneously.^5^

Alkyl chains in this size regime possess complicated potential energy landscapes that develop with chain length in fascinating ways, especially in the presence of the aromatic π cloud, which can engage in dispersive attractions with the alkyl chain.^6^–^8^ In the pure *n*-alkanes, the dihedral angles about each C–C single bond prefer *trans* (180°) over *gauche* (±60°) arrangements due to steric effects.^9^ As a result, the all-*trans* conformations are lowest in energy up to *n* = 17 ± 1,^10^ with each *gauche* ‘defect’ causing a destabilization of about 2.5 kJ mol^–1^.^11^ However, counteracting these steric effects are the dispersive interactions between sections of the alkyl chains. In a recent spontaneous Raman study of the pure alkanes by Suhm and co-workers,^10^ they showed that beyond *n* = 17 ± 1, the alkyl chains prefer to loop back on themselves.^10^ This fold is composed of four *gauche* defects separated by a single *trans* dihedral (g_*i*–2_, g_*i*–1_, t, g_*i*+1_, g_*i*+2_) that neatly place the two all-*trans* portions of the chain in an anti-parallel arrangement, commensurate with one another, as predicted by theory.^9^,^12^

A main goal of the present work is to establish when such folding first energetically competes with extended chains in the alkylbenzenes. The presence of the phenyl ring at one end of the alkyl chain provides an electron-rich π cloud to interact with the alkyl chain through attractive dispersion forces that are likely to be somewhat greater than between two alkyl chain segments.^13^,^14^ We seek to understand (i) what the nature of the fold is in this context, (ii) how it is similar to, or different from, that in the pure alkanes, and (iii) at what length alkyl chain such a fold begins to compete.

The conformations of reasonably unstrained pure *n*-alkyl chains are distinguished from one another by specifying a series of dihedral angles that are either *trans*/*anti* (180°), or *gauche* (±60°). Both the *trans* and *anti* designations are used somewhat interchangeably in the literature. We use *trans* here, in deference to the early IR work of the Strauss group on the conformations of the pure alkanes.^15^ As the length of the alkyl chains increases, naming individual conformers by labelling every dihedral angle becomes cumbersome, so we will employ the naming scheme used by the Suhm group in their study of the pure alkanes.^10^ Each conformer of a given alkylbenzene will be characterized by its deviation from the out-of-plane all-*trans* structure. Every observed conformation places C(1) in-plane and C(2) nearly perpendicular to the plane of the ring, as it is in ethylbenzene. We designated this initial dihedral as perpendicular, or p, in the previous study on short chain alkylbenzenes,^6^ so we begin numbering the dihedrals starting with the next dihedral (C_ph_–C(1)–C(2)–C(3)). Only the dihedrals that deviate from the out-of-plane all-*trans* structure will be specified. For example, the pgt conformer of *n*-butylbenzene will be referred to simply as g1, while the pgg conformer becomes g1g2 in this short-hand notation.
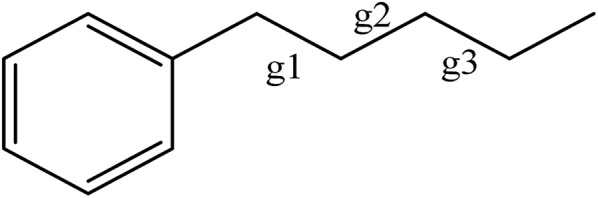


The relative sign of the *gauche* angles is also important; for this the notation g′# is used to denote when a *gauche* dihedral angle has the opposite sign of the initial g1 dihedral angle. Finally, if there is no deviation from the out-of-plane all-*trans* structure it will simply be denoted as all-*trans*.

Here, we present conformational assignments for the main observed conformers of *n*-pentyl, *n*-hexyl, *n*-heptyl, and *n*-octylbenzene, and a partial assignment of *n*-decylbenzene. In arriving at these assignments, we utilize a combination of methods, including (i) relative energies deduced from high-level theory, (ii) LIF spectra, (iii) single-conformation alkyl CH stretch IR spectra, and (iv) Raman spectroscopy, all carried out on jet-cooled samples of the molecules in the gas phase. Each of these methods brings complementary insights regarding the conformational assignments.

As we shall see, in octylbenzene, a small electronic origin transition appears in the LIF spectrum at 37 488 cm^–1^, shifted distinctly to the red of those found in smaller members of the alkylbenzenes. We present evidence that this transition arises from a g1g3g4 conformer that folds the *n*-octyl chain back over the phenyl ring where it can interact optimally with the π cloud *via* multiple CH_2_(*n*) groups.

An essential tool in interpreting the single-conformer IR spectra is the local mode Hamiltonian model developed in the Sibert group, and applied recently to ethyl, *n*-propyl, and *n*-butylbenzene.^6^ This model explicitly takes into account the strong and pervasive stretch–bend Fermi resonances that make comparison with harmonic normal mode calculations nearly impossible. We use many aspects of the model from that work without change, making at most minor modifications that further refine it. As we shall see, the method is capable of capturing many of the main characteristics of the alkyl CH stretch spectra, greatly aiding assignments. They also help in interpreting the source of unique spectral signatures when they appear. Indeed, a particular strength of the method is its ability to provide useful physical insight regarding the local site frequencies of the CH oscillators and their inter-group coupling as a function of their local configuration and position along the chain. We therefore return near the end of the manuscript to consider what that model deduces about the vibrational signatures of the alkyl chains and to assess the prospects and limitations of alkyl CH stretch spectroscopy as a tool for making structural deductions on alkyl chains.

## II. Methods

### A. Experimental

The experimental setups utilized for the UV, IR, and Raman spectroscopies have been described elsewhere,^10^,^16^ and we will only describe the specific conditions employed in this particular study. The UV and IR spectra were taken on samples entrained in 3 bar of helium and pulsed through an 800 micron orifice into a vacuum, creating a supersonic jet expansion. The samples were heated from room temperature up to 110 °C in order to sufficiently vaporize the samples. The Raman spectra were taken of samples heated from 43 °C to 81 °C and flowed continuously through a slit nozzle (0.15 mm × 4 mm) with a backing pressure of 0.5 bar of He, at a distance of 1 mm from the laser.

### B. Theoretical

Conformational equilibrium distributions for the *n*-alkylbenzenes have been calculated on a MMFF (Merck Molecular Force Field) level using Spartan'08 v.1.2.0.^17^ All conformers within a threshold of 10 kJ mol^–1^ relative to the global minimum structure were re-optimized and their energies and frequencies have been calculated with the B3LYP-D3BJ/def2TZVP method in Gaussian 09.^18^ The def2TZVP basis set represents a good compromise between computational cost and reliable results as bigger basis sets were not feasible for *n*-alkylbenzenes with an alkane chain longer than 5 carbon atoms. Previous studies have provided evidence that this level of theory provides good correspondence with experiment in ordering the relative energies of conformers.^19^ Harmonic vibrational frequency calculations were carried out on the full series of alkylbenzenes studied, from ethylbenzene to decylbenzene, at the same level of theory.

Due to the pervasive presence of strong Fermi resonance coupling between CH stretch fundamentals and the overtones of the CH scissors modes, the harmonic vibrational frequencies cannot be used in making assignments. As a result, theoretical IR spectra were calculated in a local mode basis that explicitly accounted for stretch–bend coupling, much as was done in the previous work on smaller chain alkylbenzenes.^6^ For the alkyl chains, the model Hamiltonian was constructed from the Hessian matrix and dipole derivatives computed at the B3LYP-D3/6-311++G(d,p) level of theory at each conformer's local reoptimized geometry. The only difference between the Hamiltonian construction in this work and that of the shorter chain study is how the stretch–stretch couplings between CH stretches on different carbon atoms are treated. Although the scaling of this parameter was considered in the previous work, the presence of the phenyl ring altered the local mode stretching site frequencies of the initial CH_2_ groups to the extent that there was still about a 15 cm^–1^ variance from carbon to carbon. In contrast, the local mode site frequencies for CH stretches in the middle of the longer chains in *trans* conformations are nearly degenerate, as will be seen below. The importance of the coupling between these near-degenerate sites grows with the length of the chain. In order to improve the agreement between the experimental spectrum and the theoretical model, the DFT couplings between stretches on different carbon atoms are scaled by a factor of 0.85. This adjustment appears to be transferable from chain to chain and also improves agreement between experiment and theory in the full set of conformers studied here. The use of this additional scaling on the shorter chains does not significantly alter their model spectra, as expected given the small number of near degeneracies.

## III. Results and analysis

### A. Review of short-chain alkylbenzenes

**Fig. 1:**
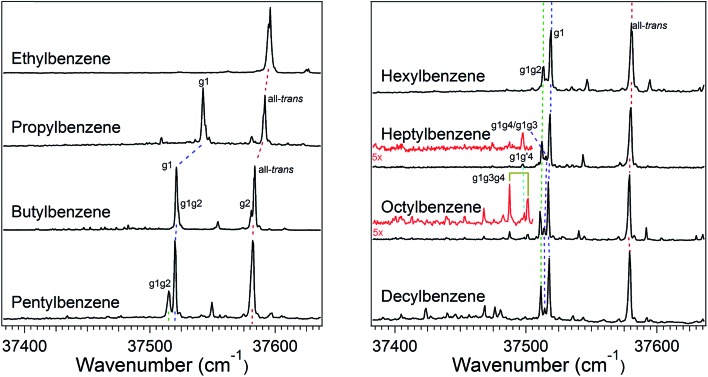
UV excitation spectra for ethylbenzene through octylbenzene, and decylbenzene. Assignments are made here for clarity, with the dashed lines indicating bands with the same assigned structure between molecules. The low frequency regions of heptylbenzene and octylbenzene are magnified 5× for clarity (red). The band located between the origins of the g1 and all *trans* conformers is a vibronic band of the g1 conformer.

The increase in conformational complexity with growing alkyl chain length is apparent even in these short-chain alkylbenzenes, with S_0_–S_1_ origin transitions for one, two, and four conformers observed and assigned for ethyl, propyl, and butylbenzene,^20^–^24^ respectively (Fig. 1). The frequency of the S_0_–S_1_ origin transition for individual conformers is especially sensitive to the first dihedral angle in the alkyl chain; *i.e.*, g1 *versus* t1. For instance, in butylbenzene, the difference in frequency between the all-*trans* and g1 conformers is approximately 62 cm^–1^, with g1 lower in frequency than the all*-trans* structure due to the interaction of the CH_*n*_(3) group with the π cloud in the g1 configuration. Single *gauche* defects at other positions along the chain cause much smaller shifts in the UV excitation frequency. Thus, the g1g2 S_0_–S_1_ origin of butylbenzene is shifted only ∼1 cm^–1^ higher in frequency than the g1 S_0_–S_1_ origin, while the S_0_–S_1_ origin of the g2 conformer is ∼2 cm^–1^ lower in frequency than the all-*trans* origin. These transitions appear as partially resolved side bands in Fig. 1, but have been better resolved in earlier studies by Simons *et al.*^20^

The relative intensities of the S_0_–S_1_ origin transitions in the LIF spectrum of butylbenzene correlate in a straight-forward manner with the relative energies for the four lowest-energy conformers of the molecule, calculated at the DFT B3LYP-D3BJ/def2TZVP level of theory. The zero-point corrected relative energies for the all-*trans*, g1, g1g2, and g2 are 0.0, 0.1, 2.1, and 2.4 kJ mol^–1^, respectively, consistent with the experimental spectrum (Fig. 1c) showing large transitions due to all*-trans* and g1, and two weaker transitions of about equal intensity due to g1g2 and g2. This shows that relative energy calculations at this level of theory can guide the choice of potential candidates for assignments.

### B. LIF spectra and relative energy calculations

The LIF spectra for pentyl, hexyl, heptyl, octyl, and decylbenzene are compared with those for the short alkylbenzenes in Fig. 1. The labels in Fig. 1 were derived through the full set of techniques brought to bear on this series of molecules. The increasing complexity of the conformational landscape for the long-chain alkylbenzenes is readily apparent in the steadily increasing number of transitions in the S_0_–S_1_ origin region with increasing alkyl chain length. This is not surprising, since with each additional carbon-atom added to the alkyl chain, another dihedral angle is introduced about which nominally t, g+, and g– configurations are possible, resulting in a geometric growth in the number of possible conformers.

**Fig. 2:**
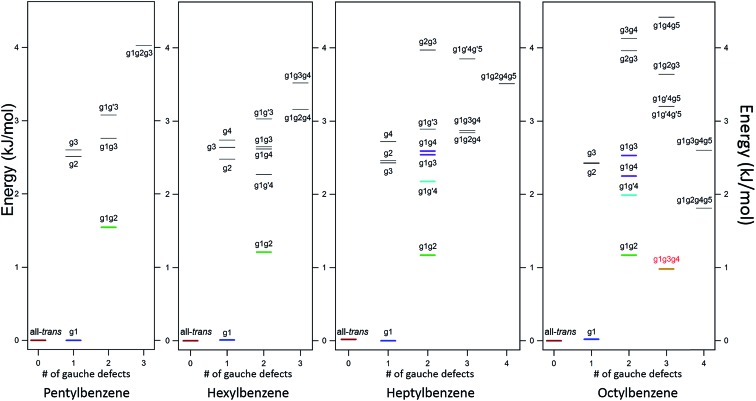
Energy level diagrams of pentylbenzene through octylbenzene. Calculations were done at the B3LYP-D3BJ/def2TZVP level of theory. The experimentally observed structures are shown with colored markers for clarity. The first observed folded structure, g1g3g4 in octylbenzene, is shown in red.

Fig. 2 shows the relative, harmonically zero-point corrected energies of conformers for pentyl, hexyl, heptyl, and octylbenzene calculated to be within 5 kJ mol^–1^ of the global minimum at the DFT B3LYP-D3BJ/def2TZVP level of theory. The all-*trans* structure is the lowest energy conformer for each of the molecules in this series, and all of the energies shown are relative to this global minimum. The energy level diagrams for each molecule are divided into sub-sets with an increasing number of *gauche* ‘defects’ going from left to right. The steady increase in the number of low-energy conformers is obvious from the figure, with 8, 11, 15, and 17 structures within the first 5 kJ mol^–1^ in pentyl through octylbenzene. In looking at the whole series of alkylbenzenes, the calculations clearly predict that the g1 conformer is always nearly isoenergetic with the all-*trans* conformer, thereby bringing C(3) above one edge of the aromatic ring, where it is stabilized by a CH···π interaction. The low energy cost of this g1 defect propagates through the conformers containing two or more *gauche* defects, with all the lowest-energy conformers possessing a g1 defect. For this reason, we have not calculated exhaustively the full set of non-g1 conformers in the larger alkylbenzenes.

The energy level diagram for pentylbenzene, then, predicts that the all-*trans* and g1 conformers should possess similar starting populations in the expansion, with the g1g2 structure third most stable. Consistent with this prediction, the LIF excitation spectrum in the S_0_–S_1_ origin region of pentylbenzene in Fig. 1 shows two intense bands (appearing at approximately the same frequencies as the g1 and all-*trans* bands for propylbenzene and butylbenzene), and a third transition which has half the intensity of larger two bands, appearing 5 cm^–1^ below the g1 origin, tentatively assigned to g1g2. Based on the results on butylbenzene, one might anticipate a fourth conformer, g2, to be present. However, the energy of the g2 conformer is raised to 2.5 kJ mol^–1^ in pentylbenzene, and so is predicted to be smaller in intensity than g1g2. We anticipate, based on butylbenzene, that the S_0_–S_1_ origin transition of g2 will be close to the all*-trans* origin, since its first dihedral angle is *trans*. It seems plausible that the g2 origin (and g3 as well) is not resolved in pentylbenzene, appearing underneath the contour due to the all-*trans* conformer, a conjecture that we will return to later.

The low-energy conformers of hexylbenzene are predicted to have a similar energy ordering to their counterparts in pentylbenzene, with all-*trans*, g1, and g1g2 minima noticeably more stable than all others. The LIF spectrum for hexylbenzene reflects this close similarity with pentylbenzene, also displaying two large transitions due primarily to all-*trans* and g1 conformers, and a weaker-intensity band tentatively assigned to g1g2 just to the red of the g1 origin. At higher energies, the energy diagram is modified by the presence of new conformations arising from the additional dihedral associated with the lengthening of the alkyl chain (*e.g.*, g1g′4, g1g4, g1g2g4, and g1g3g4). Notably, the hexyl chain in hexylbenzene is the shortest chain that can support a g1g3g4 conformer, with its chain folded back over the ring, here with calculated energy of 3.52 kJ mol^–1^ above the global minimum. As we shall see, there is no experimental evidence for this conformer in hexylbenzene.

The LIF spectrum of heptylbenzene differs from those of pentyl and hexylbenzene in two subtle but important ways. First, the gap in frequency between the bands tentatively assigned to g1 and g1g2 electronic origins has now widened just enough to show a small peak half way between them, at about one-third the size of the g1g2 origin. Second, a weak transition appears at 37 497 cm^–1^, some 15 cm^–1^ red of the g1g2 origin. The carrier of this band must have a stronger interaction of the heptyl chain with the aromatic π cloud than do other conformers, leading to the additional red-shift in its origin. Therefore, there are five resolved electronic origins to account for *via* the spectroscopy.

The energy level diagram for heptylbenzene (Fig. 2) is similar to that of pentyl and hexylbenzene. The g1g′4 conformer is fourth in energy and therefore a candidate for one of the new transitions. The next conformer up in energy with prospects for appearing near to or red-shifted from the g1 origin is g1g3. Further distinction between these possibilities will require the infrared and Raman data in the following sections. Note that there is a slight drop in energy of the g1g3g4 structure with the longer heptyl chain, but several other folded or partially folded structures compete with it in energy.

In octylbenzene, the LIF spectrum now more clearly shows the three transitions near the g1 origin. Notably, there are several small transitions red-shifted from the UV transitions of the g1-like conformers, including a band with intensity similar to the central peak of the g1 triad. We shall present evidence shortly that this band is due to the g1g3g4 conformer, consistent with a precipitous drop in its relative energy to a value slightly below that of the g1g2 conformer (Fig. 2).

Finally, Fig. 1 presents an overview LIF scan of decylbenzene. While we have not studied this molecule in detail, we present its spectrum as a part of the series, since it shows a number of red-shifted transitions now appearing with significant intensity, indicating the growing presence of more conformers with strong interactions of the chain with the aromatic π cloud.

### C. Fluorescence-dip infrared spectra

**Fig. 3:**
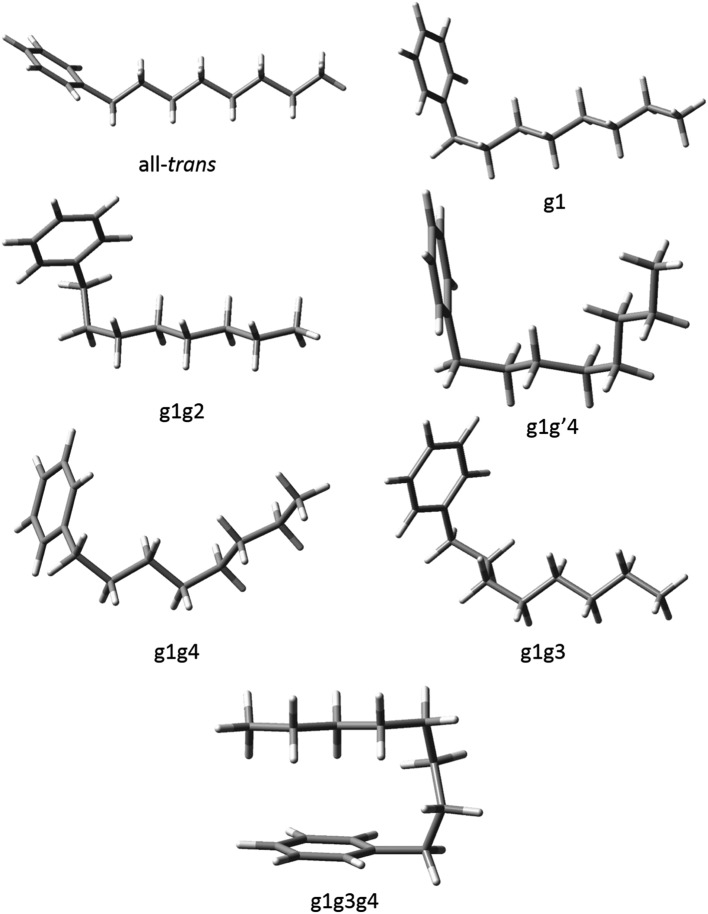
The seven experimentally observed conformations seen in the alkylbenzene series, shown using octylbenzene as the model system.

Using fluorescence-dip infrared spectroscopy (FDIRS), infrared spectra in the C–H stretch region (2800–3000 cm^–1^) were taken of the individual conformers of pentylbenzene, hexylbenzene, heptylbenzene, octylbenzene, and a subset of the main bands of decylbenzene. As we have argued in the preceding section, the LIF spectra evolve with lengthening alkyl chain in a systematic way that enables us to sort the conformational isomers of each sized alkylbenzene into conformational families that share the same or closely analogous alkyl chain conformations. In this section, we confirm that these similarities carry over to the single-conformation IR spectra, which are fit using the local mode Hamiltonian model described in Sec. II.B. Using octylbenzene as representative, we present optimized structures for every conformational family that we discuss in the sections that follow (Fig. 3). The labels on the peaks in Fig. 1 indicate the assigned structures based on the full complement of methods used. Given the importance of the folded g1g3g4 conformer, we present and discuss this structure separately.

**Fig. 4:**
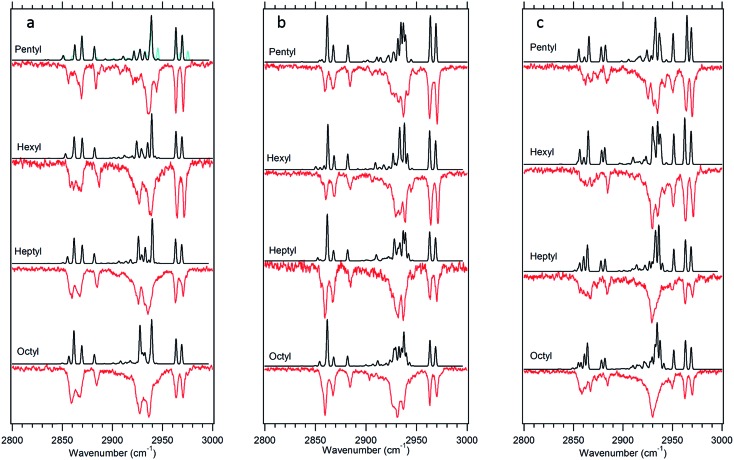
(a–c) Experimental infrared spectra of pentylbenzene through octylbenzene for the all-*trans* (a), g1 (b), and g1g2 (c) conformers (red) compared to the calculated spectra for the same conformations (black). The blue spectrum on the all-*trans* pentylbenzene carries some weight from the g3 conformer (3 : 1 all-*trans* : g3 ratio).

**Fig. 5:**
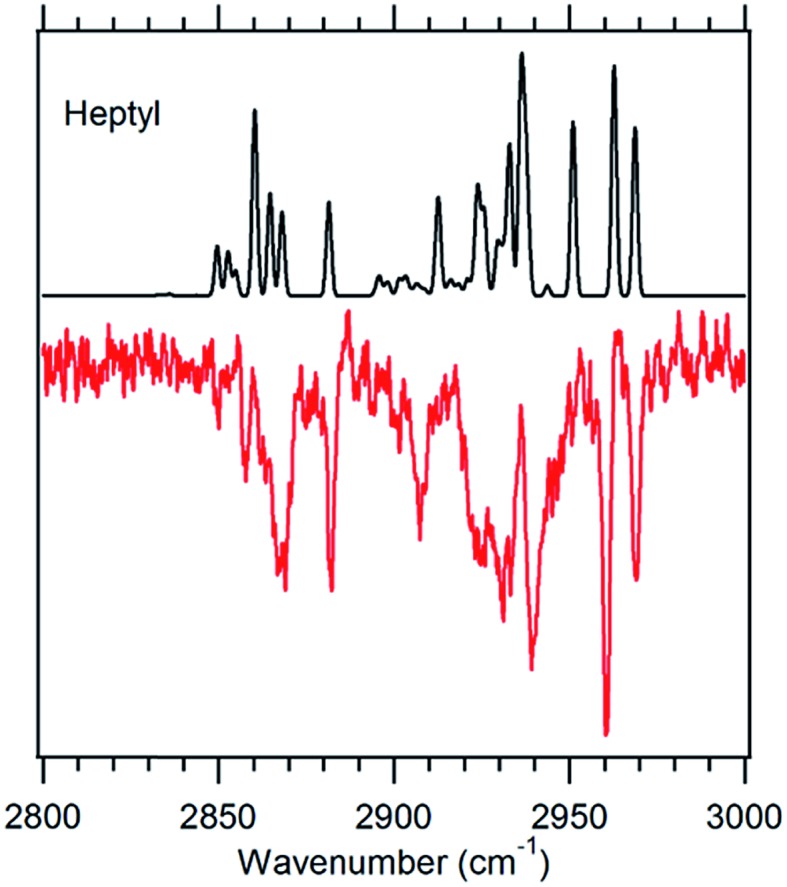
g1g'4 theoretical spectrum (black) *versus* the experimental spectrum of heptylbenzene taken at 37 498 cm^–1^ (red).

**Fig. 6:**
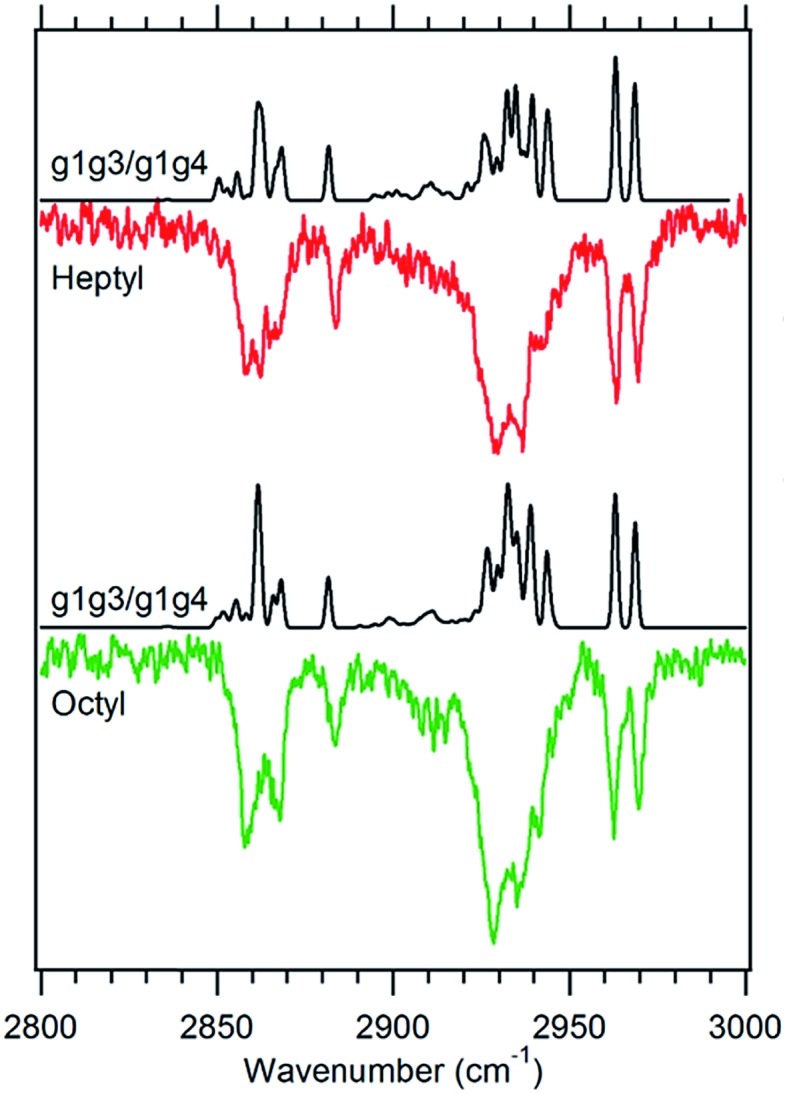
Theoretical spectra of a 1 : 1 ratio of the g1g3 and g1g4 conformers (black) *versus* the experimental spectra of heptylbenzene (red) and octylbenzene (green) taken at 37 514 cm^–1^.

**Fig. 7:**
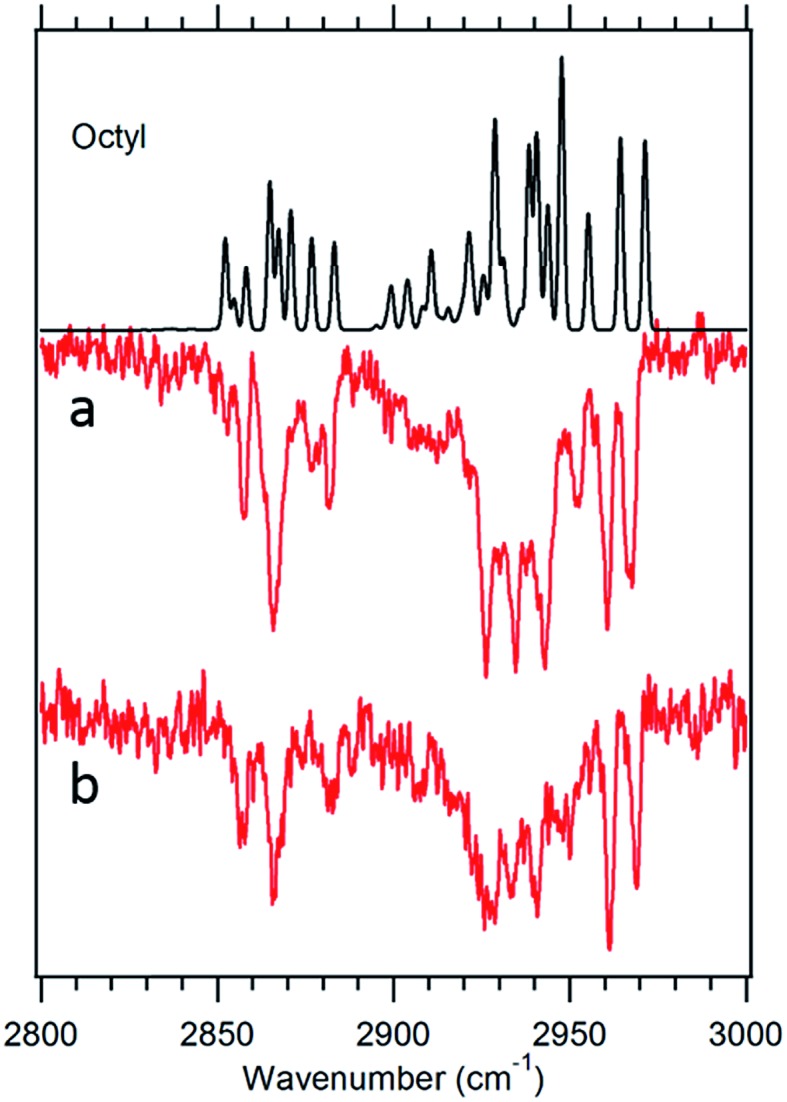
Theoretical spectrum of the g1g3g4 conformer (black) *versus* the experimental spectra of octylbenzene taken at 37 488 cm^–1^ (a) and 37 502 cm^–1^ (b).

The experimental single-conformation spectra of each sized alkylbenzene in the series are grouped by family in Fig. 4–7. The theoretical prediction of the local mode Hamiltonian model is placed above each experimental spectrum. These model results will be used in the Discussion section to analyze the changes in spectra with conformation and chain length. Here, we use these results primarily as a means of determining and/or strengthening the conformational assignments. There are certain common features of all spectra worth mentioning at the outset, to orient the reader. In every spectrum, the doublet in the 2960–2970 cm^–1^ region is the near-degenerate pair of asymmetric stretch transitions of the CH_3_ group. These bands change very little from one sized alkylbenzene to the next. A weak band at 2883 cm^–1^ is also common to all spectra, assigned to the methyl symmetric stretch. These methyl transitions decrease in relative intensity down the series for the simple reason that the number of CH_2_ groups in the chain grows with alkyl chain length, adding to the integrated intensity of transitions due to the CH_2_ groups. The spectral regions due to the CH_2_ groups that make up the alkyl chain contribute intensity primarily in two regions, 2850–2890 and 2920–2950 cm^–1^. The lower wavenumber region is primarily due to the CH_2_ symmetric stretches/Fermi resonances, while the higher region contains both asymmetric CH_2_ stretch and the upper members of the symmetric stretch/Fermi resonances. As the length of the chain grows, when the alkyl chain is in a conformation in which many CH_2_ groups are in similar environments, their site frequencies will be similar, and coupling between CH_2_ groups can lead to normal modes extended over several CH_2_ groups, with intensities governed by the relative phases of oscillations of the groups involved. On the other hand, if the local configuration and environment of a given CH_2_ group is sufficiently distinct, as it could be in the presence of one or more *gauche* defects, the local mode site frequencies of each CH can shift, leading to partial localization of the modes.

#### All-*trans* and g1 conformers

The out-of-plane all-*trans* and g1 conformers (Fig. 3) are the two dominant conformers present in the LIF spectra of every molecule in the series. The UV S_0_–S_1_ origin bands for these two conformers are located at roughly the same frequency for each molecule in the series (Fig. 1), and are the most intense bands in every UV spectrum. Fig. 4a and b present single-conformation IR spectra in the 2800–3000 cm^–1^ region of the all-*trans* and g1 bands of pentyl through octylbenzene, respectively.

The series of all-*trans* spectra show the development of the fully-extended alkyl chain conformation with chain length. These spectra differ from those in the g1 family in fairly subtle ways, not surprisingly given the fact that a single *gauche* defect in g1 leads to longer and longer tracts of *trans* dihedrals as the alkyl chain length grows. The asymmetric methyl CH stretch transitions appear as a doublet at 2963 and 2969 cm^–1^ in the spectra of both conformers. The series of spectra in the two families differ most noticeably in the 2850–2870 cm^–1^ region, where both have a set of two experimentally resolved bands due to the CH_2_ groups whose relative intensity differences are accurately predicted by the anharmonic model calculations. The calculations also match the developments of the spectra in the middle region of each spectrum (2920–2950 cm^–1^) with good accuracy. The quality of the overall fits is sufficient to give confidence to the assignments of the two main bands in the LIF spectra to the all-*trans* and g1 conformers. As we can see, small differences between experiment and theory are accounted for, at least in part, by small contributions from other minor conformers whose UV transitions overlap with these main bands (Fig. 4a).

#### g1g2 conformers

The S_0_–S_1_ origin bands tentatively assigned to the members of the g1g2 conformational family are the third largest transitions in each of the UV spectra, appearing just below the g1 origin, and slightly lower in frequency (–9 cm^–1^) than the assigned g1g2 origin in butylbenzene. The infrared spectra for the g1g2 conformers, shown in Fig. 4c, are in many ways similar to those of their all-*trans* and g1 counterparts. However, the g1g2 spectra differ in two characteristic ways from those in Fig. 4a and b. First, a new band appears at 2950 cm^–1^, with an intensity that is relatively strong at shorter chain lengths, and somewhat weaker in longer chains, as would be expected of a CH_2_ transition. The frequency of this transition is shifted into the gap between the main asymmetric stretch CH_2_ transitions and the methyl doublets. This band is also present in the theoretically predicted spectra, arising principally from the first CH_2_ group in the alkyl chain, the benzylic CH_2_. Second, the symmetric stretch for this same benzylic CH_2_ group is similarly shifted to higher frequency than its counterparts, producing a third band, that is weak, but clearly visible in the experimental spectra at ∼2874 cm^–1^, just below the CH_3_ symmetric stretch band. The theoretical model also predicts the presence of this shift, but slightly overestimates its magnitude. These characteristic features of the infrared spectra of the g1g2 conformer provide confidence in the assignment of the g1g2 conformer to the third major band in the UV spectra (Fig. 1).

#### g1g′4 conformer

Heptylbenzene is the shortest molecule in the series for which a band lower in frequency than the g1g2 S_0_–S_1_ origin band (Fig. 1) is present with enough intensity that its infrared spectrum could be taken. As the conformer next in energy after g1g2 (Fig. 2), the g1g′4 conformer (Fig. 3) is a leading candidate for the carrier of this spectrum. The FDIR spectrum recorded while monitoring fluorescence from this band (Fig. 5) looks generally similar to those observed for the previous conformers: the CH_3_ stretch fundamentals are present at the same frequencies, there is a significant buildup of bands in the 2920–2950 cm^–1^ region due to the CH_2_ asymmetric stretches, and there are three main peaks in the low frequency region of the spectrum due to the symmetric stretches. The most significant difference between the g1g′4 spectrum and that of the previous conformers is the presence of the band at 2910 cm^–1^. No analogous strong band is present in the spectra of other conformers, making the transition a signature of the g1g′4 conformer, as the g1g′4 theoretical spectrum is the only calculated spectrum with a band in this region.

The model calculations in Fig. 5 show a reasonable correspondence with experiment, although the intensity patterns are not fully captured by the model. For instance, the theoretical spectrum predicts a strong band at 2950 cm^–1^, which is present in octylbenzene's spectrum, but appears only as a shoulder in heptylbenzene. Despite these deficiencies, the unique nature of the band at 2910 cm^–1^, when combined with the predictions of the energy calculations, suggests the tentative assignment of the g1g′4 structure to the 37 498 cm^–1^ band of heptylbenzene. In octylbenzene, the corresponding transition closest in UV wavelength to its partner in heptylbenzene was not recorded due to its weak intensity. As we shall see shortly, the band immediately to the blue is a vibronic band of the g1g3g4 turn.

#### g1g3/g1g4

The fifth unique S_0_–S_1_ origin transition observed in the alkylbenzene series is the small peak located at 37 515 cm^–1^ between the g1g2 and g1 UV S_0_–S_1_ origins (Fig. 1). This UV transition is first observed in hexylbenzene, but the first molecule in which it is sufficiently isolated for FDIR spectroscopy is heptylbenzene. The infrared spectra for this band in both heptylbenzene and octylbenzene are shown in Fig. 6, showing their close similarity. Unfortunately, the FDIR spectra lack much by way of unique transitions, preventing a completely firm assignment. Given the close proximity of the electronic origin to the g1 and g1g2 origins, we anticipate that the conformer of interest has g1 as its first dihedral, with only weak additional interactions with the aromatic ring. The relative energy calculations (Fig. 2) predict that the g1g3 and g1g4 conformers are next highest in energy. Indeed, the calculated spectra in Fig. 6 contain equal contributions from g1g3 and g1g4, leading to a reasonable fit with experiment.

#### The first folded conformer: g1g3g4

As noted previously, the LIF spectrum of octylbenzene presents a unique S_0_–S_1_ origin, which is shifted to the red of all others appearing at 37 488 cm^–1^, –92 cm^–1^ from its all-*trans* counterpart. This shift suggests a stronger interaction of the alkyl chain with the aromatic ring. At the same time, the B3LYP-D3BJ/def2TZVP calculations predict that the energy of the g1g3g4 conformer drops to within 0.98 kJ mol^–1^, putting it lower in energy than the g1g2 conformer (Fig. 2). The FDIR spectrum of this band in octylbenzene is shown in Fig. 7a, where it is compared with the predictions of the theoretical model. The experimental spectrum shows several unique features that set it apart from others. First, the number of resolved bands in both symmetric stretch and asymmetric stretch regions is significantly greater than in other conformers. Second, a strong transition appears at 2950 cm^–1^, which is faithfully reproduced by the calculations. This band has been associated with adjacent *gauche* defects, as is indeed the case in g1g3g4. The theoretical model also correctly predicts the presence of three main bands in the 2920–2950 cm^–1^ region, as well as the weak bands in the 2900–2920 cm^–1^ region, which are unresolved but clearly present in the experimental spectrum. Finally, the theory correctly predicts the much larger number of unique transitions in the 2850–2880 cm^–1^ region, where other conformers had only three symmetric stretch bands. When combined, the close agreement of the theoretical model with the experimental spectrum, the prediction that g1g3g4 is now a low-energy conformer, and the shift of the electronic origin well below that of all other conformers, provide the basis for a firm assignment of this band to the g1g3g4 conformer of octylbenzene. As Fig. 3 shows, this conformer has the alkyl chain folded back over the aromatic ring where it can interact with it strongly, stabilizing its energy and leading to its unique spectral characteristics in both the UV and IR.

Fig. 7b presents the analogous spectrum of the band in octylbenzene at 37 502 cm^–1^, shown in the inset of Fig. 2. Its spectrum is identical to that at the g1g3g4 origin, consistent with it being a vibronic band, appearing due to Franck–Condon activity in a low-frequency mode involving motion of the alkyl chain against the π cloud, as one might anticipate following π–π* excitation.

### D. Raman spectroscopy

**Fig. 8:**
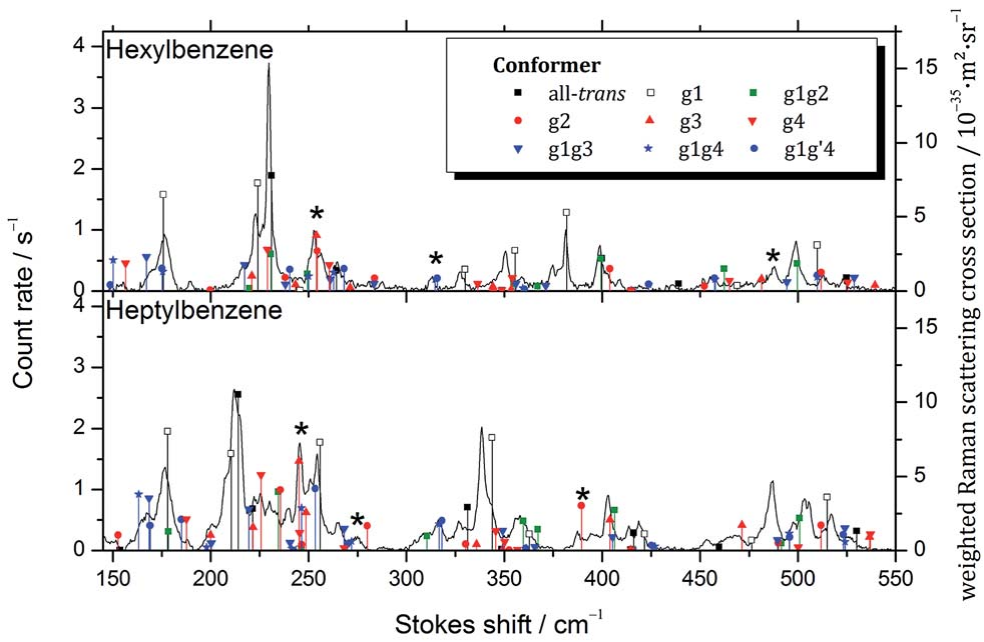
Raman spectra of hexylbenzene and heptylbenzene shown with unscaled calculated stick spectra for the low energy conformations weighted by their calculated relative abundance at 298 K. Bands assigned to conformers not observed in the UV/IR studies are marked with asterisks.

While considering the LIF spectra in Section III.B, we noted the potential for conformers such as g2 or g3 to have S_0_–S_1_ origins in the UV that are unshifted from those of the all-*trans* parent, and therefore would be inaccessible to study without interference from the all-*trans* counterpart, which is significantly more highly populated. In order to test this conjecture more directly, Raman spectra of the low frequency Stokes region of hexylbenzene through decylbenzene were recorded in Göttingen, recognizing that the low-frequency vibrations are likely to be more readily uniquely identified. Importantly, these spectra are not conformation-specific, containing contributions from all conformers present in the expansion. The resulting Raman spectra over the 150 to 550 cm^–1^ vibrational excitation region for hexylbenzene and heptylbenzene are presented in Fig. 8, with the corresponding spectra of octyl and nonylbenzene included in ESI.† In all cases, comparison is made between the experimental spectra and stick spectra that report the relative Raman intensities and unscaled wavenumber positions for the full set of low energy conformers of each molecule, calculated at the same DFT B3LYP-D3BJ/def2TZVP level of theory used throughout this manuscript. Calculated Raman activities *A*_k_ and depolarization ratios *P*_k_ were converted into parallel and perpendicular polarization fractions of the scattered light and the former attenuated by a factor of 1.5 + *ν̃*_k_···0.0002 cm^–1^ due to the empirical transmission properties of the monochromator. From this corrected Raman activity *A*cork, Raman scattering cross sections were calculated according to ref. 10. An average vibrational temperature of 100 K was assumed for hot band contributions. The cross sections were further weighted by estimated abundances based on rigid rotor harmonic oscillator Gibbs energies at 298 K, assuming that the population was not relaxed substantially from that in the nozzle.

The calculated stick spectra of the three lowest energy conformers, all-*trans*, g1, and g1g2, provide remarkably accurate predictions for most of the major bands observed in hexylbenzene; however, there are a few bands, notably those around 255, 315, and 485 cm^–1^, marked with asterisks in the figure, that are not accounted for using only the three major conformers. These bands are accounted for if we include the g1g′4 structure, as well as the single *gauche* conformers g2 and g3. This lends credibility to the idea that the S_0_–S_1_ origin bands for some of the single *gauche* conformers, g2 and g3, are present as minor conformers in the supersonic free jet, with S_0_–S_1_ origin transitions unshifted from their all-*trans* counterparts.

Heptylbenzene shows this same trend, requiring the single *gauche* conformers g2 and g3 for the assignment of multiple bands in the Raman spectrum (*e.g.*, 245, 275, 387 cm^–1^). There is some argument to be made for the g1g3g4 conformer providing the best match with a couple of weak bands in this heptylbenzene spectrum; however, there are no strong bands that require it, and as such we do not claim its presence in heptylbenzene.

The Raman spectra for octylbenzene and nonylbenzene are shown in the ESI (Fig. S1 and S2†). The spectral congestion associated with the increasing chain length makes it progressively more difficult to make firm conformational assignments of many of the weak bands in the Raman spectrum. However, in octylbenzene, there is some evidence for folded structures like g1g3g4 being present, most notably, in the region around 275 cm^–1^ and at 420 cm^–1^, albeit with low intensity.

## IV. Discussion

The primary motivation for this work was to examine the conformational preferences of longer alkyl chains linked to an aromatic ring in the prototypical case of the *n*-alkylbenzenes. Using a combination of LIF excitation spectra, single-conformation IR spectra in the alkyl CH stretch region, Raman spectroscopy, and dispersion-corrected DFT calculations, our results on pentyl through octylbenzene have shed light on three important issues: (i) the evolution of the conformational landscape of alkylbenzenes as the alkyl chain length increases, (ii) the earliest appearance of a folded structure in straight chain alkylbenzenes, and (iii) the spectroscopic signatures and CH stretch site frequencies of specific conformations, as revealed by the anharmonic local mode Hamiltonian model used to assign the spectra.

### A. Conformational preferences of the alkyl chain in alkylbenzenes

#### General features of the conformational landscape

In this series of alkylbenzenes, we see a steady increase in the number of resolved conformations as the chain length increases. The UV spectrum is itself a sensitive indicator of the interactions of the chain with the phenyl ring: the all-*trans* conformer (Fig. 3) has its S_0_–S_1_ origin furthest to the blue in the S_0_–S_1_ UV spectrum (Fig. 1), while the g1 conformer, with a single *gauche* defect about the first alkyl chain dihedral, results in a red-shift of 62 cm^–1^ in its origin band, almost independent of the length of the all-*trans* alkyl chain that follows this *gauche* defect. These two electronic origins are nearly the same size, indicating similar populations, and consistent with the B3LYP-D3BJ calculations, which show the g1 conformer nearly isoenergetic with all-*trans*. By contrast, in pure alkane chains, isolated *gauche* defects raise the energy of the chain by ∼2.5 kJ mol^–1^. As Fig. 2 shows for octylbenzene, CH_2_(3) engages in a stabilizing CH···π interaction, which affects the UV and lowers the energy of g1 so that this first *gauche* defect can occur with no energetic penalty.

*Gauche* defects that occur at positions further down the alkyl chain are energetically more costly. As expected based on the pure alkanes, the single-*gauche* conformers g2, g3, *etc.*, are 2.5–3.0 kJ mol^–1^ less stable than the all*-trans* and g1 conformers. The energy level diagrams in Fig. 2 are divided into sub-groups differing in the number of *gauche* defects, with the lowest energy structures with 2, 3, and 4 *gauche* defects in heptylbenzene having energies 1.2, 2.9, and 3.6 kJ mol^–1^ less stable than the global minimum, indicating a degree of stabilization of more tightly folded structures.

Over the entire pentyl- to octylbenzene series, the g1g2 conformer is third lowest in energy, appearing 1.2–1.5 kJ mol^–1^ above the global minimum (green line in Fig. 2). Its S_0_–S_1_ origin appears –6 cm^–1^ from the g1 origin, shifted slightly due to the reorientation of CH_2_(3) in accommodating a second *gauche* dihedral of the same sign. The stabilization of adjacent *gauche* defects of the same sign is also characteristic of pure alkanes, and is referred to as the ‘positive pentane effect’, since it appears first in pentane. By contrast, g1g′2 has a calculated energy 5.6 kJ mol^–1^ above the global minimum, destabilized by 4.0 kJ mol^–1^ compared to g1g2 (and therefore above the energy cut-off in Fig. 2) due to steric effects between C_*i*_ and C_*i*+4_, where C_*i*_ is the first carbon involved in the *gauche* defects, when adjacent *gauche* defects have opposite sign. In the infrared, the g1g2 conformer possesses a characteristic transition at 2950 cm^–1^, showing up in the gap between the CH_2_ asymmetric stretch (AS)/symmetric (SS) FR peaks (2920–2940 cm^–1^) and the methyl AS doublet (2963/2969 cm^–1^).

Beginning with hexylbenzene, the g1g′4 conformer (Fig. 3) is next in energy (2.3 kJ mol^–1^, Fig. 2), stabilized slightly compared to its g1g4 counterpart, which is at an energy (2.7 kJ mol^–1^) expected of single *gauche* defects in pure alkyl chains. The slight stabilization of g1g′4 relative to g1g4 is opposite to that found in next-nearest-neighbor *gauche* defects (*e.g.*, g1g′3 higher in energy than g1g3), suggesting that the g1g′4 alkyl chain gains additional stabilization from dispersive interactions with the π cloud, perhaps transmitted from C(3) to C(6) (see Fig. 2).^7^

#### The g1g3g4 fold in octylbenzene

Arguably the most important finding of the present work is the detection and characterization of the g1g3g4 fold, first appearing with measurable intensity in octylbenzene. The work of Suhm and co-workers on pure, straight-chain alkanes presented evidence of a folded structure utilizing a (g_*i*–2_, g_*i*–1_, t, g_*i*+1_, g_*i*+2_) hairpin motif beginning at approximately *n* = 17/18, with hairpin structures quickly becoming the dominant conformational motif above *n* = 20.^10^ In all of the straight-chain alkanes with *n* < 20, the fully extended all-*trans* structure is the main conformational motif. In order to maximize dispersive attractions between the two legs of the hairpin, the fold occurs at or near the center of the chain, so that the two legs are nearly equal in length.

The alkylbenzene series appears to follow the same trend, with extended structures with either no *gauche* defect (all-*trans*) or a single *gauche* defect about the C(1)–C(2) bond (g1) dominating the conformational distribution up to heptyl and octylbenzene. The LIF spectra of undecylbenzene and dodecylbenzene (Fig. S3, ESI†) show significantly increased absorption intensity in transitions to the red of the main bands, where folded structures are anticipated to appear, suggesting a rapid increase in the population of folded structures similar to that observed in the pure alkanes.

The shortest chain alkylbenzene in which a folded structure is observed is significantly shorter than the first observed folded structure of the pure alkanes, with the alkylbenzenes first folding at a chain length of 8 (octylbenzene), approximately half the length of the first fold in the pure alkanes (Fig. 8). There are two main differences between the alkylbenzenes and alkanes that result in the earlier folding in the former case.

**Fig. 9:**
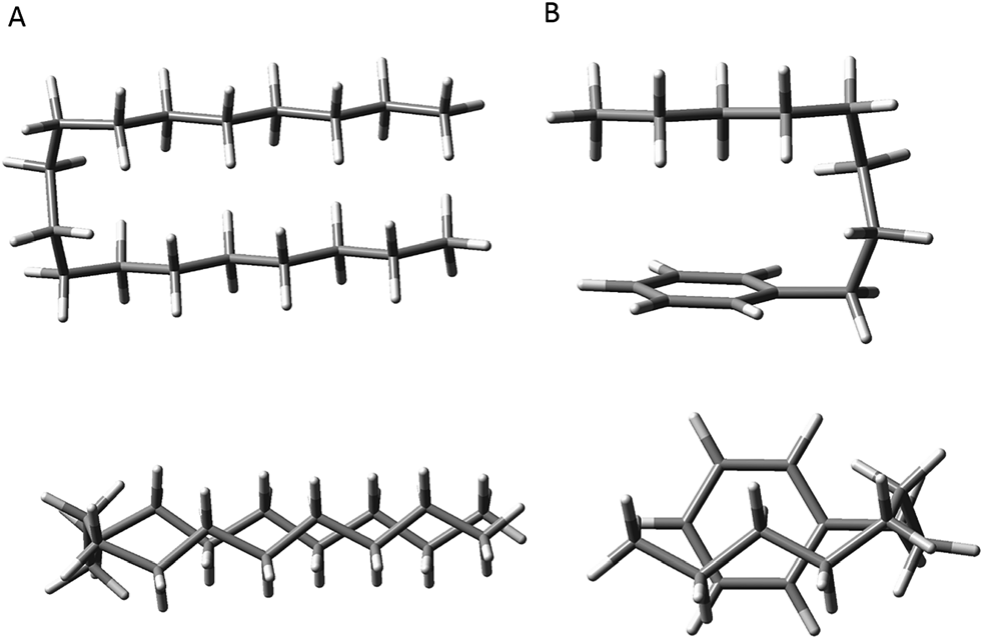
Comparison of the hairpin turn observed in the pure alkanes by the Suhm group (A) *versus* the turn observed in octylbenzene (B).

First, the energetic cost of the turn is significantly reduced when it begins immediately adjacent to the phenyl ring. The reader will recall that in pure alkanes, relative to an all-*trans* structure, each *gauche* defect raises the energy by approximately 2.5 kJ mol^–1^, with the four-*gauche* turn thereby requiring up to 10 kJ mol^–1^, due to the pentane effect. This destabilization is compensated by the stabilization associated with the dispersive attractions between the all*-trans* segments on either side of the turn, which the turn brings into perfect alignment with one another, as shown in Fig. 9a. In octylbenzene, the g1g3g4 turn has the C(*ortho*)–C(ph)–C(1)–C(2) dihedral nominally perpendicular. However, its actual value is 67°, so that a more proper label might be 'g0g1g3g4' if we label this dihedral ‘0’ using our nomenclature, which is quite close to its pure alkane ideal of 60°. Thus, it is in effect the same turn as in the pure alkanes, as shown in Fig. S4,† where the two structures are overlaid on one another. Since the out-of-plane orientation of the alkyl chain is common to all low-energy structures of the alkylbenzenes, the ‘g0’ defect is not actually a defect, but a distinct preference. Furthermore, the energy of the g1 conformer is nearly isoenergetic with all-*trans*, indicating that the C(ph)–C(1)–C(2)–C(3) dihedral occurs at no cost in energy, due to the stabilizing interaction of CH_2_(3) with the aromatic π cloud (see Fig. 3). The two remaining *gauche* defects, g3 and g4, are on adjacent carbons and are of the same sign, reducing their energy slightly relative to non-adjacent defects. In essence, the conformational energy penalty is reduced by a factor of two compared to alkanes.

Second, formation of the turn brings the alkyl chain back over the aromatic ring where it can experience dispersive interactions with the π cloud at C(5)–C(8), as shown most clearly in the top view of the structure in Fig. 9b. In this sense, the phenyl ring substitutes for one leg of the all-*trans* chains, but provides a set of CH···π type C···C interactions that are stronger than those between alkyl chains. In addition, the aromatic ring itself is less restrictive in its requirements on the turn, providing a wider swath of angles for stabilization of the single alkyl chain.

As Fig. 2 shows, the g1g3g4 conformer drops from 3.6 kJ mol^–1^ above the global minimum in hexylbenzene to 2.9 kJ mol^–1^ in heptyl-, and on down to 1.0 kJ mol^–1^ in octylbenzene, where it is first clearly observed experimentally. This makes clear that the stabilization provided by the ring is a cumulative effect due to interactions that extend beyond C(6), which is nearly ring-center, to C(7) and C(8) that interact primarily with the edges of the ring (Fig. 9b).

#### Entropy effects and equilibrium populations

One initially puzzling aspect of the identification of the g1g3g4 conformer in octylbenzene is the weak intensity of the transitions in the LIF spectrum ascribed to this conformer, given its calculated relative energy only 1.0 kJ mol^–1^ above the global minimum, nearly isoenergetic with the g1g2 conformer. While there are many reasons that could contribute to lack of correspondence between S_0_–S_1_ origin intensity and the fractional abundances (*e.g.*, differences in Franck–Condon activity, or S_1_ fluorescence quantum yield), these appear to be minor in the alkylbenzenes in that low-frequency vibronic activity is typically small, and measured S_1_ lifetimes are nearly identical. Contributions from conformers whose electronic origin transitions are unresolved also needs to be taken into account, but changes the results only modestly. Even after taking account of these effects and the unusually intense low-frequency Franck–Condon progression in g1g3g4 (marked by a tie line in Fig. 1), the integrated intensity of the g1g3g4 conformer is surprisingly small given its low relative energy. This is also confirmed by the Raman spectra up to nonylbenzene (ESI†), which do not show major contributions from g1g3g4, either.

However, the tight fold of the g1g3g4 conformer leads to an entropic penalty that significantly reduces its equilibrium population at the pre-expansion temperature. Indeed, as Table S5 in ESI† shows, the relative Gibbs free energy of g1g3g4 at 298 K, Δ*G*(298), is 6 kJ mol^–1^ above the all-*trans* free energy global minimum. If the barriers separating conformers of the alkyl chain are large enough relative to *kT* (2.48 kJ mol^–1^ at 298 K), the pre-expansion populations will be essentially frozen in during the cooling in the expansion. We have calculated select barriers involving isomerization about single C–C bonds, and find them to be in the 12–15 kJ mol^–1^ range (about 5*kT*), and consistent with the work of Klauda *et al.*^25^ on pure alkanes. Thus, we anticipate that the downstream populations in the expansion will partially reflect the equilibrium populations prior to expansion.

### B. Interpreting the alkyl CH stretch spectra *via* the model

One of the particular strengths of the anharmonic local mode Hamiltonian model used to model the single-conformer alkyl CH stretch infrared spectra is that it provides a ready means of extracting physical insight regarding the major factors contributing to unique IR bands appearing in the spectra. A first level of understanding develops simply from consideration of the uncoupled site frequencies for each of the CH stretch oscillators in the alkyl chains. These appear as diagonal elements in the Hamiltonian matrix that contains entries for each of the CH fundamentals and scissors overtones and combination bands.

#### Site frequencies and spectroscopic signatures in the all-*trans* conformers

**Fig. 10:**
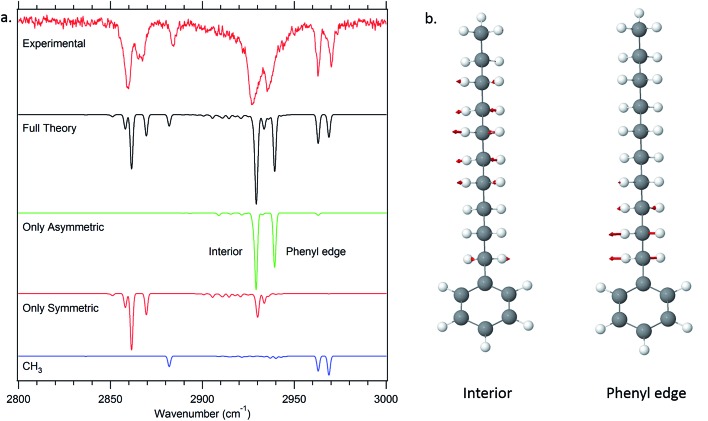
Dipole decomposition of the theoretical spectrum of decylbenzene (a). The two major delocalized asymmetric modes of the all-*trans* conformer of decylbenzene are displayed as well (b).

The high symmetry of the alkyl chains in the all-*trans* conformers makes them a natural starting point for interpretation *via* the model, since they give rise to particularly simple spectra (Fig. 4a) with a common set of transitions whose intensities change smoothly with increasing alkyl chain length. We have also recorded the FDIR spectrum of the all-*trans* conformer of *n*-decylbenzene, shown in Fig. 10a, since this long chain has nine CH_2_ groups that more nearly reflect the long-chain limit for the all-*trans* series. One initially puzzling aspect of the spectrum of decylbenzene is the presence of two dominant transitions in the symmetric stretch asymmetric stretch region (2920–2940 cm^–1^) rather than the single transition expected of an infinite all-*trans* chain. Deeper insight into the origin of each of the peaks can be found *via* a dipole decomposition, much as was done for propyl and butylbenzene spectra in earlier work.^6^ In those cases, the dipole decompositions were obtained by computing a series of model spectra in which only one CH_2_ or CH_3_ group's local mode stretches were allowed to carry oscillator strength. However, since the all-*trans* conformer of *n*-decylbenzene contains numerous consecutive CH_2_ groups that are nearly equivalent, a dipole decomposition that examines one CH_2_ group at a time misses the important constructive and destructive interferences between groups that lead to the final spectrum.

Thus, in the all-*trans* conformers, we selectively extract components due to the symmetric or asymmetric stretches rather than individual CH_2_ groups. The dipole decomposition in these terms is shown in Fig. 10a, in which the two intense features in the 2920–2940 cm^–1^ region are primarily due to two bright asymmetric stretch fundamentals. Since the asymmetric stretches are not Fermi-coupled, the nature of these two bright states can be elucidated from this more nearly normal mode picture. The higher-frequency asymmetric stretch bright state involves contributions primarily from the two CH_2_ groups immediately adjacent to the phenyl ring (Fig. 10b), with diminishing in-phase contributions from the remaining CH_2_ groups further down the chain. The vibration responsible for the other bright state, about 25 cm^–1^ lower in frequency, comes from the in-phase asymmetric stretches from the remaining set of CH_2_ groups further down the chain remote from the phenyl ring or CH_3_ group. These stretches are out of phase with the phenyl-adjacent CH_2_, ensuring orthonormality of the states. We see then, that the primary reason that there are two bright asymmetric combinations instead of one (as would be anticipated in a simple one-dimensional Hückel model) is due to edge effects. The phenyl ring raises the local mode frequencies of the first two CH_2_ groups sufficiently to lead to a splitting of the bright states in two.

**Fig. 11:**
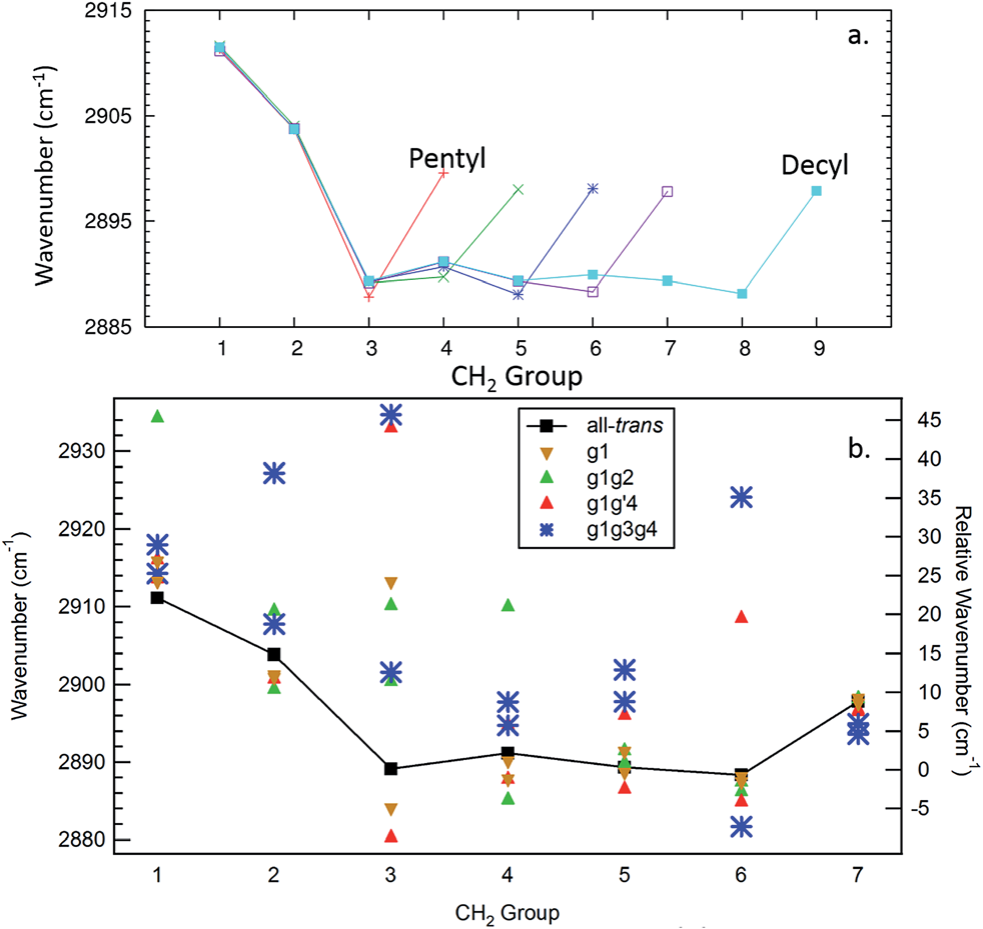
Site frequencies for the all-*trans* conformers (a) and the observed conformers of octylbenzene (b).

The site frequencies in the all-*trans* CH_2_ groups as a function of position relative to the phenyl ring are shown in Fig. 11a for the full set of alkylbenzenes of interest here, from *n*-pentyl through *n*-decylbenzene. Since the two CH groups on each carbon atom are in equivalent positions in the all-*trans* conformers, a single site frequency is associated with each CH_2_(*n*). The edge effects are immediately apparent from the figure. On the phenyl ring end of the alkyl chain, the benzylic CH_2_(1) and CH_2_(2) are shifted to higher wavenumber to 2912 and 2904 cm^–1^, respectively, +23 and +15 cm^–1^ above the value of the central CH_2_ groups in the chain (2889 cm^–1^). Similarly, the methyl group affects a single CH_2_ group immediately adjacent to it, which is characteristically at 2898 cm^–1^, a +9 cm^–1^ shift. Thus, in the alkylbenzenes, the number of CH_2_ groups unaffected by edge effects is three shorter than the CH_2_ total and four shorter than the number of carbon atoms in the alkyl chain as a whole (*e.g.*, 6 CH_2_ groups in decylbenzene).

The difference in site frequencies depicted in Fig. 11a translates directly into the degree of delocalization of the asymmetric CH_2_ stretch fundamentals, splitting up the alkyl chain into the pair nearest to the phenyl ring, the central CH_2_ groups, and the single CH_2_ adjacent to the terminal CH_3_ group. Coupling between CH_2_ groups on the two edges is negligible, while even minor inter-CH_2_ coupling leads to delocalization of the vibrations in the unperturbed central group. The all-*trans* conformers thus enable refinement of the magnitude of the scaling stretch coupling between different carbons, as mentioned in the Theoretical methods section. In the all-*trans* conformer of *n*-pentylbenzene, there is a single ‘central’ CH_2_(3), and the four AS CH_2_ modes divide into the benzylic coupled pair (which constructively interfere to dominate the spectrum), and two single CH_2_ groups at lower wavenumber. As the number of nearly equivalent CH_2_ groups grows, the bright AS CH_2_ band at lower frequency grows in intensity, as anticipated, until it dominates the spectra for the longest alkyl chains, as shown in Fig. 10a for *n*-decylbenzene. Similar arguments hold for the bright pair of symmetric stretch fundamentals (2862 and 2869 cm^–1^), with the added complication of the Fermi resonances with the scissors overtones.

### C. Site frequencies and spectroscopic signatures for *gauche* defects and the g1g3g4 turn

Fig. 11b presents the analogous local mode site frequencies for individual CH oscillators of the g1, g1g2, g1g′4 and g1g3g4 conformers of octylbenzene. The all-*trans* results are included in the graph, connected by a solid line, in order to facilitate ready comparison with this limiting case. The g1 conformer follows the same trends as in all-*trans*, except for the CH group involved in the CH···π interaction (CH(3)), which is shifted up in frequency by 23 cm^–1^ to 2913 cm^–1^, a value close to that in CH_2_(1). This frequency increase has been noted in several other contexts in which CH groups are involved in CH···X interactions with an electron-rich acceptor (*e.g.*, O, N, halogen), commonly referred to as a ‘blue-shifted hydrogen bond’.^7^ Here we see that this ‘blue-shift’ carries over to intramolecular interactions between an alkyl chain and an aromatic π cloud. The presence of the g1 defect thus extends the number of coupled CH groups on the phenyl end of the chain by an additional CH, but doesn't otherwise shift the frequencies of the bright CH stretch modes appreciably. This leads to spectra for the g1 conformers that are surprisingly similar to their all-*trans* counterparts (Fig. 4b).

The g1g2 conformer is the first and lowest-energy example of a structure in which two adjacent *gauche* defects are present. In this conformer, the CH(1) oscillator on the same side as the all-*trans* segment of the carbon backbone, has its vibrational site frequency pushed up to 2935 cm^–1^, +46 cm^–1^ above the typical mid-chain all-*trans* CH_2_ (2889 cm^–1^). This large shift can largely be attributed to a steric effect between the two CH stretches on C1 and C4, respectively. The distance between these H atoms is about 2.31 Å. In the all-*trans* case, the phenyl-adjacent local mode site frequencies are both 2911 cm^–1^, so the steric interaction between the C1 and C4 CH stretches shifts the frequency by an additional 24 cm^–1^ on the C1, and a corresponding shift up to 2910 cm^–1^ is seen on its steric partner. This leads to a characteristic IR transition at 2950 cm^–1^ that is the clearest spectral signature of the presence of adjacent *gauche* defects.

The g1g3g4 turn structure shows several characteristic spectroscopic signatures that set it apart from all other conformers of octylbenzene, including a well-resolved band at 2955 cm^–1^, a triad of peaks in the CH_2_ AS region, greater absorption intensity in the 2900–2920 cm^–1^ region, and a set of five well-resolved transitions in the CH_2_ SS region. While most of the transitions are mixtures of contributions from several CH_2_ groups, the site frequencies for g1g3g4, shown in Fig. 11b, reflect the unique environments of the CH_2_ groups in the turn structure and contribute to a greater degree of localization of the CH_2_ stretch modes than in the non-turn structures. The unique site frequencies are most notable in the C(1)–C(3) series involved in the turn, where one of the CH groups is pushed up in frequency from 2918 to 2927 to 2935 cm^–1^. The 2935 cm^–1^ site frequency is a result of the strong CH···π interaction of CH_2_(3), and contributes most (coefficient of 0.73) to the 2955 cm^–1^ transition in the full spectrum. The site frequency of CH_2_(2) is shifted up in frequency due to a C(1)–C(2) dihedral of 65.7°, and is primarily responsible for the strong band at 2947 cm^–1^ in the full spectrum. Similar to the effects in the g1g2 spectra, the site frequency is shifted up due to steric interactions. Here the shortest H–H distance is between the C(2) and C(5) sites at 2.16 Å. Interestingly, both of the C(5) site frequencies are shifted up from their all*-trans* conformer value of 2890 cm^–1^; the C(5) CH stretch that is close to the C(2) is shifted up to 2902 cm^–1^, but the other site frequency associated with the CH stretch that points away from the ring is also shifted up to 2898 cm^–1^. Importantly, CH_2_(6) is also shifted to high frequency (2924 cm^–1^), and contributes to the middle band of the CH_2_ AS triad. This shift to higher frequency arises from a second strong CH···π interaction, since CH_2_(6) sits directly over the middle of the phenyl π cloud. This shift contributes to a localization of the CH_2_ vibrations in the remaining *trans* segments of the alkyl chain (CH_2_(4), CH_2_(5), CH_2_(7)), which also leads to several prominent, distinct features in the 2900–2920 cm^–1^ region, similar to what the localization did for the signature peak in the heptylbenzene g1g′4 conformer. Several of the features discussed here for the g1g3g4 turn are reflected in extra CH Raman intensity for folded structures observed for the longer pure alkanes, in particular between 2900 and 2950 cm^–1^ (Fig. 11 in ref. 10).

## V. Conclusions

The present study used a combination of electronic frequency shifts, single-conformer alkyl CH stretch infrared spectra, dispersion-corrected DFT energies, and low-frequency Raman spectra to assign the observed UV transitions to specific conformers of alkylbenzenes in the size range from pentyl to octylbenzene. The FDIR spectra presented an opportunity to apply and refine the local-mode based anharmonic model of the alkyl CH stretch/scissor overtone region.

The alkylbenzenes are challenging molecules on which to apply single-conformation spectroscopy, since the IR-UV double resonance methods used require unique UV transitions to obtain truly conformation-specific IR spectra. The S_0_–S_1_ origins are sensitive to the first dihedral, but increasingly insensitive to *gauche* defects further from the aromatic ring. This causes small contributions from higher-energy conformers to contribute to some of the spectra.

However, a primary goal of the present work was to identify and characterize folded chain structures in which the chain comes back over the phenyl π cloud. This additional interaction with the aromatic ring shifts the S_0_–S_1_ origin of turn structures to the red of the all-*trans* and g1 conformer origins, where single-conformer spectroscopy is possible without interference. In octylbenzene, a UV transition shifted –92 cm^–1^ from the all-*trans* origin was assigned to the g1g3g4 turn. Its infrared spectrum was unique in several respects due to the shifts to higher frequencies of several of the CH site frequencies, which led to greater localization of the CH_2_ transitions than in most other conformers.

It will be interesting to see how this and competing turn structures evolve with increasing alkyl chain length. Cursory LIF spectra of decyl, undecyl, and dodecylbenzene show a number of transitions shifted well to the red of the non-folded electronic origins, motivating future work to characterize them.

## Supplementary Material

Supplementary information

## References

[cit1] Battin-Leclerc F. (2008). Prog. Energy Combust. Sci..

[cit2] Eschner M. S., Groger T. M., Horvath T., Gonin M., Zimmermann R. (2011). Anal. Chem..

[cit3] Eschner M. S., Welthagen W., Groger T. M., Gonin M., Fuhrer K., Zimmermann R. (2010). Anal. Bioanal. Chem..

[cit4] Striebich R. C., Shafer L. M., Adams R. K., West Z. J., DeWitt M. J., Zabarnick S. (2014). Energy Fuels.

[cit5] Darcy D., Nakamura H., Tobin C. J., Mehl M., Metcalfe W. K., Pitz W. J., Westbrook C. K., Curran H. J. (2014). Combust. Flame.

[cit6] Tabor D. P., Hewett D. M., Bocklitz S., Korn J. A., Tomaine A. J., Ghosh A. K., Zwier T. S., Sibert E. L. (2016). J. Chem. Phys..

[cit7] Hobza P., Havlas Z. (2000). Chem. Rev..

[cit8] Karthikeyan S., Ramanathan V., Mishra B. K. (2013). J. Phys. Chem. A.

[cit9] Thomas L. L., Christakis T. J., Jorgensen W. L. (2006). J. Phys. Chem. B.

[cit10] Lüttschwager N. O. B., Suhm M. A. (2014). Soft Matter.

[cit11] Balabin R. M. (2009). J. Phys. Chem. A.

[cit12] Goodman J. M. (1997). J. Chem. Inf. Comput. Sci..

[cit13] Fujii A., Hayashi H., Park J. W., Kazama T., Mikami N., Tsuzuki S. (2011). Phys. Chem. Chem. Phys..

[cit14] Goursot A., Mineva T., Kevorkyants R., Talbi D. (2007). J. Chem. Theory Comput..

[cit15] Snyder R. G., Aljibury A. L., Strauss H. L., Casal H. L., Gough K. M., Murphy W. F. (1984). J. Chem. Phys..

[cit16] Ensminger F. A., Plassard J., Zwier T. S., Hardinger S. (1995). J. Chem. Phys..

[cit17] Spartan, 1.2.0, Wavefunction Inc., 1991–2013.

[cit18] Frisch M. J., Trucks G. W. T., Schlegel H. B., Scuseria G. E., Robb M. A., Cheeseman J. R., Scalmani G., Barone V., Petersson G. A., Nakatsuji H., Li X., Caricato M., Marenich A., Bloino J., Janesko B. G., Gomperts R., Mennucci B., Hratchian H. P., Ortiz J. V., Izmaylov A. F., Sonnenberg J. L., Williams-Young D., Ding F., Lipparini F., Egidi F., Goings J., Peng B., Petrone A., Henderson T., Ranasinghe D., Zakrzewski V. G., Gao J., Rega N., Zheng G., Liang W., Hada M., Ehara M., Toyota K., Fukuda R., Hasegawa J., Ishida M., Nakajima T., Honda Y., Kitao O., Nakai H., Vreven T., Throssell K., Montgomery Jr J. A., Peralta J. E., Ogliaro F., Bearpark M., Heyd J. J., Brothers E., Kudin K. N., Staroverov V. N., Keith T., Kobayashi R., Normand J., Raghavachari K., Rendell A., Burant J. C., Iyengar S. S., Tomasi J., Cossi M., Millam J. M., Klene M., Adamo C., Cammi R., Ochterski J. W., Martin R. L., Morokuma K., Farkas O., Foresman J. B. and Fox D. J., Gaussian, Gaussian Inc., 2013.

[cit19] Bocklitz S., Suhm M. A. (2015). Z. Phys. Chem..

[cit20] Dickinson J. A., Joireman P. W., Kroemer R. T., Robertson E. G., Simons J. P. (1997). J. Chem. Soc., Faraday Trans..

[cit21] Borst D. R., Joireman P. W., Pratt D. W., Robertson E. G., Simons J. P. (2002). J. Chem. Phys..

[cit22] Hopkins J. B., Powers D. E., Smalley R. E. (1980). J. Chem. Phys..

[cit23] Fujii A., Fujimaki E., Ebata T., Mikami N. (2000). J. Chem. Phys..

[cit24] Fujimaki E., Fujii A., Ebata T., Mikami N. (2001). J. Phys. Chem. A.

[cit25] Klauda J. B., Brooks B. R., MacKerell A. D., Venable R. M., Pastor R. W. (2005). J. Phys. Chem. B.

