# Supplementary material for: Identifying the first folded alkylbenzene via ultraviolet, infrared, and Raman spectroscopy of pentylbenzene through decylbenzene
Source: Chem Sci. 2017 May 23;8(8):5305–18. doi: 10.1039/c7sc02027a (PMC6223349; doi:10.1039/c7sc02027a)
Supplement: Supplementary file 1 [file SC-008-C7SC02027A-s001.pdf]

Electronic Supplementary Material (ESI) for Chemical Science.
This journal is © The Royal Society of Chemistry 2017

Supplemental Information

Identifying the First Folded Alkylbenzene via Ultraviolet, Infrared, and Raman Spectroscopy of Pentylbenzene through Decylbenzene

Daniel M. Hewett, Sebastian Bocklitz, Daniel P. Tabor,
Edwin L. Sibert III,* Martin A. Suhm, and Timothy S. Zwier*

Department of Chemistry, Purdue University, West Lafayette IN, 47907
Department of Chemistry, University of Wisconsin-Madison, Madison WI, 53706
Institut für Physikalische Chemie, Universität Göttingen, Göttingen, Germany

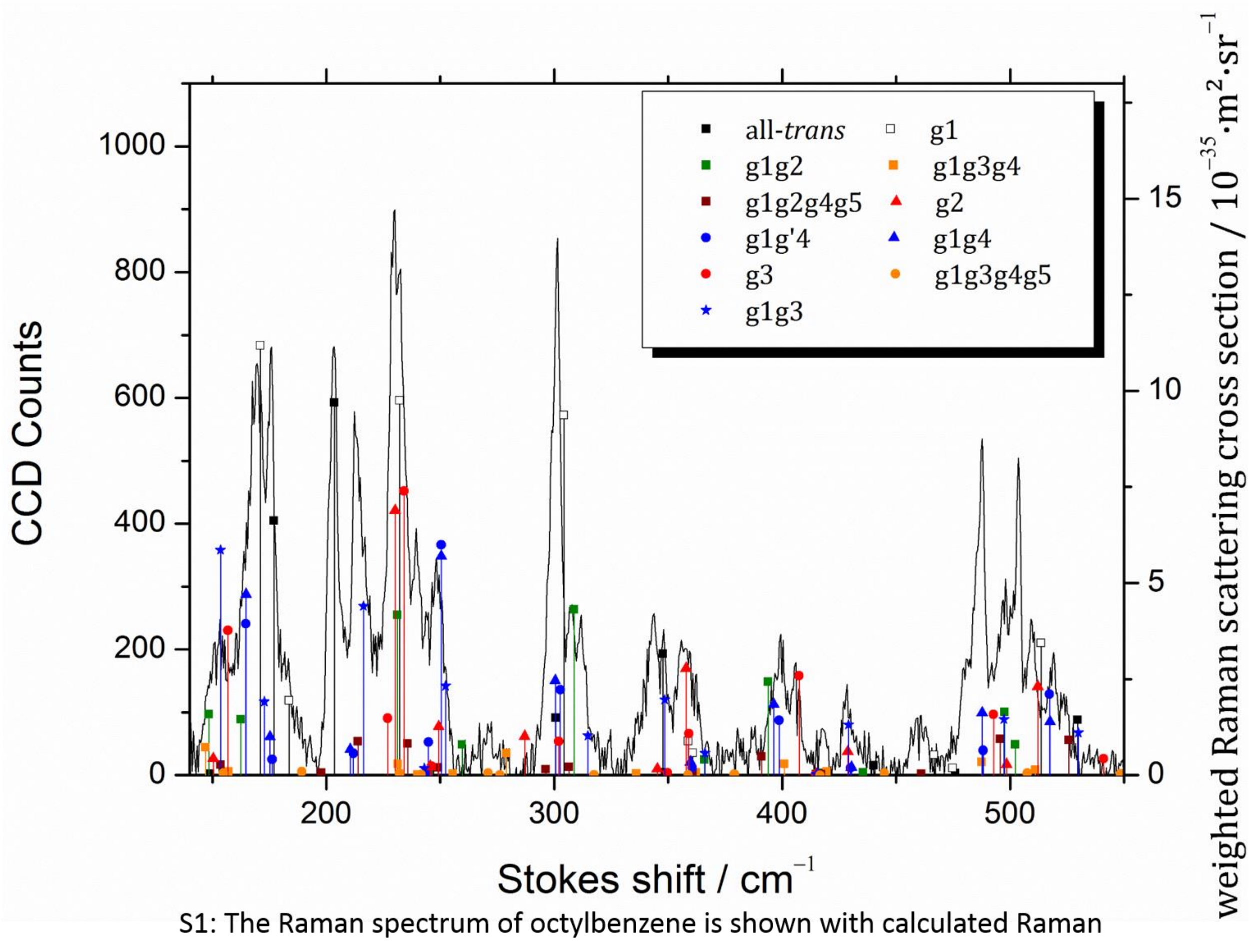

S1: The Raman spectrum of octylbenzene is shown with calculated Raman scattering cross sections scaled using the Gibbs free energies for the low energy conformations. The list of conformers included is not exhaustive, but instead uses only a selection of single, double, triple, and quadruple gauche conformations.

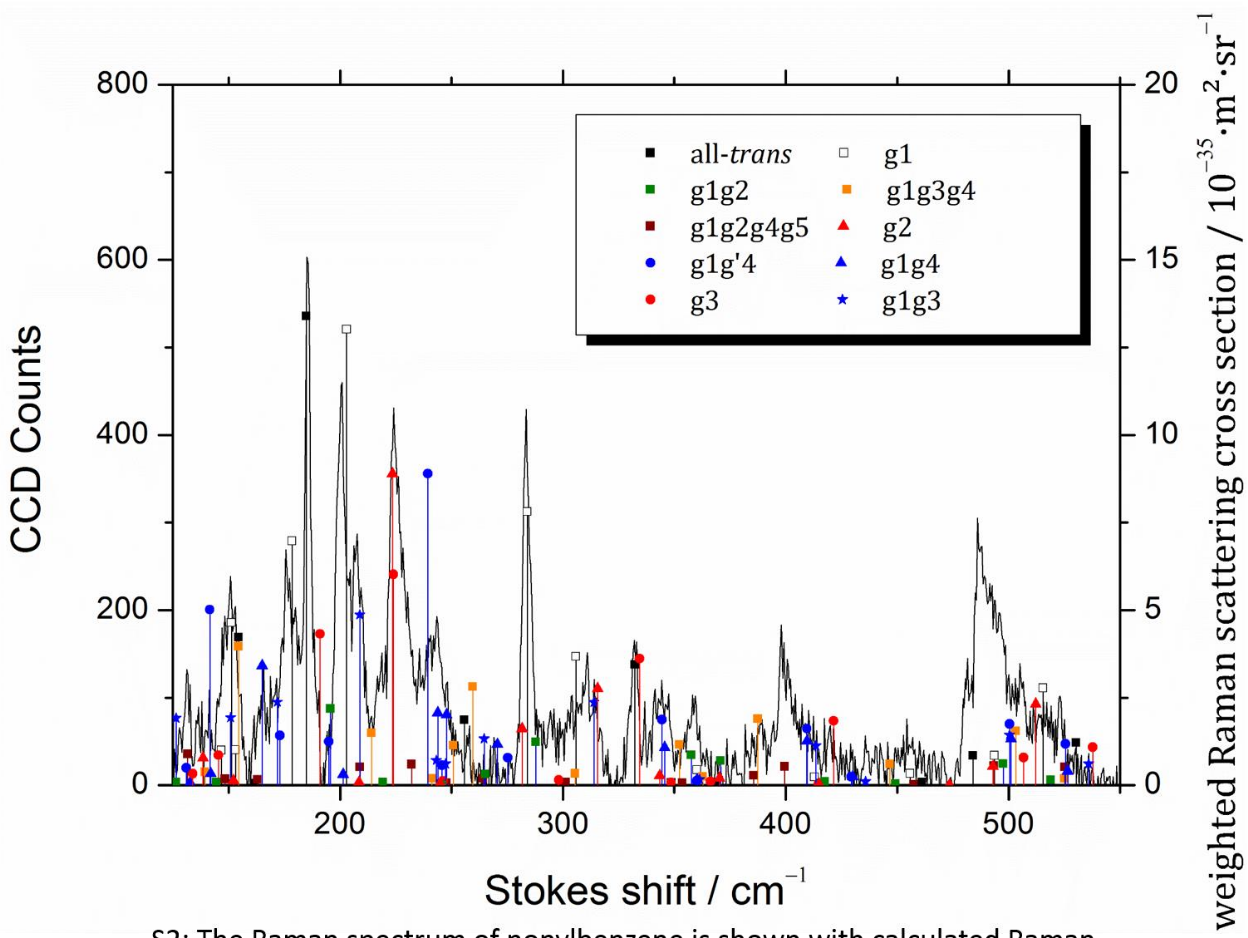

S2: The Raman spectrum of nonylbenzene is shown with calculated Raman scattering cross sections scaled using the Gibbs free energies for the low energy conformations. The list of conformers included is not exhaustive, but instead uses only a selection of single, double, triple, and quadruple gauche conformations.

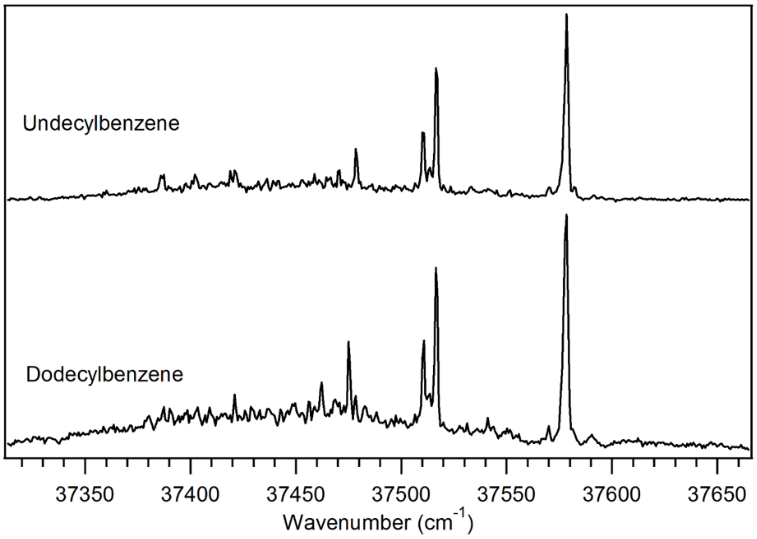

S3: UV Spectra for Undecylbenzene and Dodecylbenzene. These spectra show the rapid increase in activity below 37500 cm^{-1} as the chain length increases beyond eight carbon atoms.

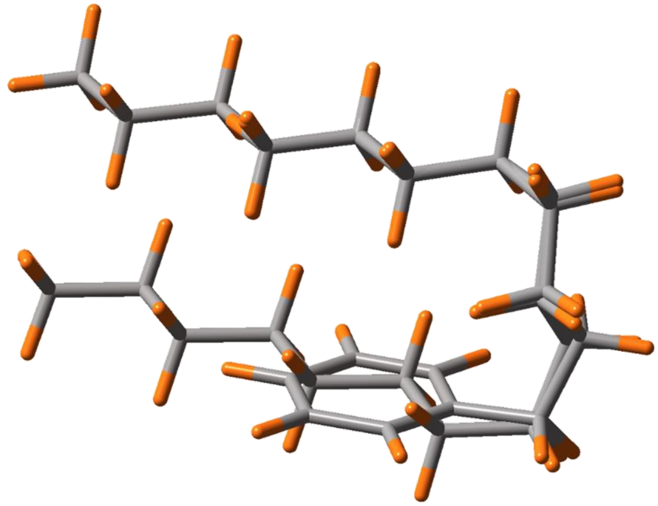

S4: Alkane folded structure overlayed with the g1g3g4 structure of octylbenzene. This overlay shows the similarity in the hairpin turns observed in both the pure alkanes and octylbenzene.

S5: Calculated Gibbs free energies for octylbenzene and nonylbenzene conformers at 298 K in the RRHO approximation.

Octyl	kJ/mol
trans	0
g1	0
g1g2	1
g1g3g4	6
g1g2g4g5	3
g1g'4	1
g1g4	1
g3	1
g2	1
g1g3	1
g1g3g4g5	7

Nonyl	kJ/mol
trans	0
g1	-1
g1g3g4	2
g1g2g4g5	5
g1g'4	1
g1g4	2
g1g3	2
g3	1
g1g2	4
g2	1
